# Multifrequency AFM integrating PeakForce tapping and higher eigenmodes for heterogeneous surface characterization

**DOI:** 10.3762/bjnano.16.142

**Published:** 2025-11-17

**Authors:** Yanping Wei, Jiafeng Shen, Yirong Yao, Xuke Li, Ming Li, Peiling Ke

**Affiliations:** 1 Public Technology Center, Ningbo Institute of Materials Technology and Engineering, Chinese Academy of Sciences, Ningbo 315201, China

**Keywords:** atomic force microscopy (AFM), high eigenmodes, multifrequency AFM, nanoscale material analysis, surface characterization

## Abstract

This study introduces a multifrequency atomic force microscopy (AFM) technique that synergistically integrates PeakForce tapping mode with higher eigenmode vibrations to achieve simultaneous high-resolution topographical imaging and to access additional contrast channels for distinguishing material regions or compositions. Unlike conventional multimodal AFM, our method employs non-resonant and higher eigenmode frequencies to achieve robust topographical and compositional mapping. Our experimental results indicate that the superposition of high-eigenmode vibrations, when applied at low amplitudes, does not significantly interfere with the topographical and nanomechanical mappings obtained via the PeakForce tapping method. Furthermore, the technique’s dual capability, that is, quantitative mechanics via quasi-static force curves and qualitative material-sensitive information via eigenmode vibration signals, facilitates effective compositional differentiation in heterogeneous nanomaterials while significantly simplifying the requirements for probe selection, which are typically necessary for material differentiation via the standard PeakForce tapping method. This innovation enhances the technique’s practicality and extends compatibility to a wider array of probe types.

## Introduction

Atomic force microscopy (AFM) has become an indispensable tool for characterizing the morphology and surface properties of materials at the micro- and the nanoscale [1–5]. Among its various operating modes, tapping mode AFM is particularly prevalent due to lateral force minimization and its ability to give phase-contrast images of heterogeneous surfaces [6]. This mode involves oscillating the cantilever near its resonance frequency with the tip interacting intermittently with the sample surface. By monitoring and controlling the cantilever’s amplitude and phase, topographical and phase images can be generated, providing insights into material properties and enabling the differentiation of regions or components within heterogeneous samples [7–9].

The advent of multimodal AFM has extended the capabilities of tapping mode, exciting the cantilever at several eigenmode frequencies simultaneously. The amplitude of the primary eigenmode serves as feedback for topographical measurements, while the higher eigenmodes enhance material contrast. These signals can be correlated with specific interaction types, such as conservative and dissipative processes [10–12]. Despite these advances, critical limitations persist. The inherent nonlinearity of tip–sample dynamics in tapping and multimodal AFM often introduces imaging artifacts. Typical examples include topographic step-like distortions and sudden phase-contrast inversions arising from bistable transitions between co-existing oscillation states, which complicate data interpretation [13–17]. Additionally, operational complexity escalates in liquid environments, where low-quality factor (*Q*) cantilever dynamics amplify noise and demand meticulous parameter tuning [18].

To overcome these limitations, PeakForce tapping mode (PFT) was developed. It employs vertical probe oscillations at subresonant frequencies (0.5–8 kHz) to establish quasi-static tip–sample contact [18–19]. Unlike traditional dynamic modes, PFT eliminates the need for intricate cantilever tuning by real-time regulation of the interaction force at each scan point. This innovation not only simplifies operation but also enables high-resolution topographic imaging with exceptional stability, even in challenging environments such as liquids [18–19].

As demonstrated by force–distance curve analyses [19], PFT enables direct extraction of quantitative mechanical properties (e.g., Young’s modulus and adhesion) for material discrimination. However, this capability is constrained by the requirement for precise probe stiffness (*k*) matching, a critical requirement for accurate contact mechanics models [20]. The stiffness *k* should be neither too large (to ensure a sufficient deflection signal for accurate force measurement) nor too small (to achieve adequate sample indentation). For heterogeneous samples with modulus variations exceeding two orders of magnitude, this necessitates iterative probe selection, complicating high-throughput characterization [21].

In this study, we introduce a hybrid multifrequency AFM technique that synergistically integrates non-resonant PeakForce modulation with eigenmode vibrations to overcome these challenges. This configuration harnesses the mechanical precision of quasi-static force control and the sensitivity of eigenmode signals to variations in surface properties and enables simultaneous topographical, mechanical, and compositional mapping. We apply this technique to montmorillonite (MMT) nanosheets, demonstrating its potential to improve material property contrast and characterization.

## Experimental

### Experimental setup

**Figure 1:**
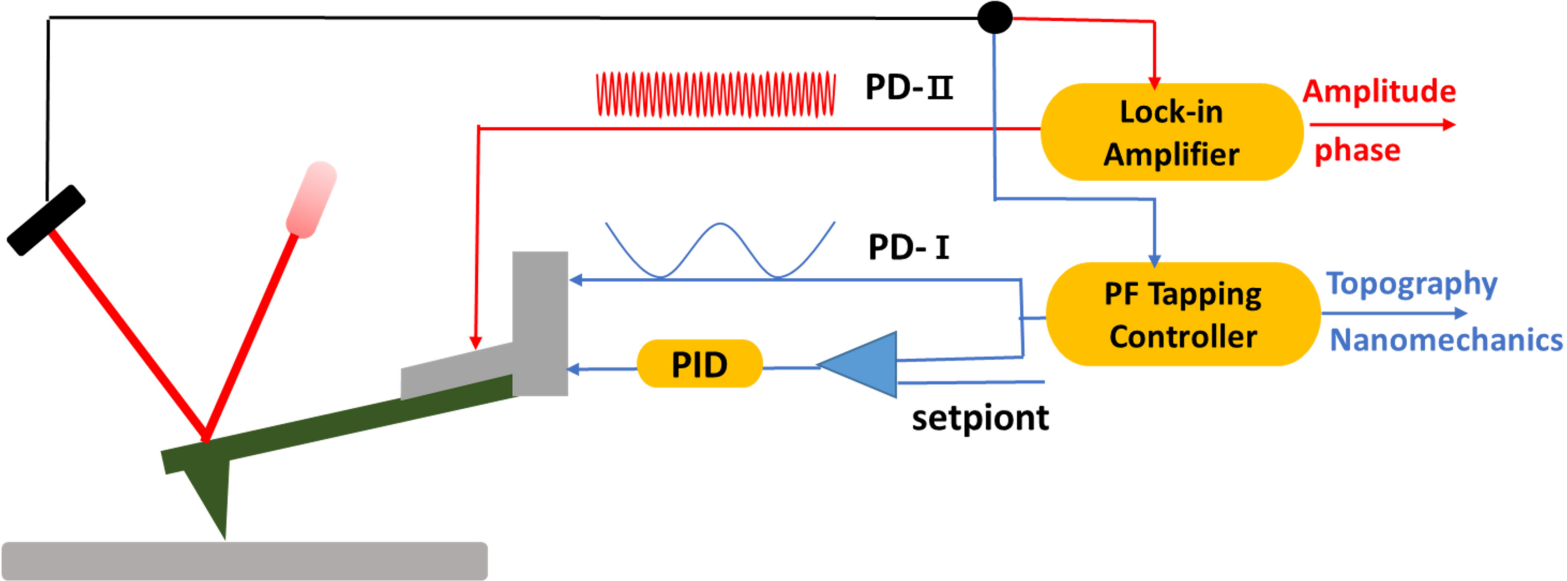
Schematic of the multifrequency AFM setup implemented on a Bruker Dimension Icon. The cantilever is excited by two independent piezos. (1) PeakForce tapping (blue path): Piezo driver PD-I imposes a low-frequency sinusoid (0.5–2 kHz, *f* ≪ *f*_1_) that cyclically brings the tip into and out of contact. The resulting force–distance curve is captured in real time; the peak force *F* is fed to the PID controller to maintain a constant set-point while the *z*-scanner records topography. Each curve is analyzed with contact mechanics models for quantitative mechanical mapping. (2) High-eigenmode path (red): Piezo actuator PD-II drives a second oscillation at the second (or higher) eigenmode. The corresponding amplitude *A* and phase φ are extracted by lock-in amplifiers for compositional contrast.

Our experiments were conducted using a commercial AFM system (Bruker Dimension Icon) equipped with a cantilever holder that incorporates a piezoelectric actuator for external excitation. We built upon the PFT mode by incorporating an additional excitation signal applied to the cantilever at its higher eigenmode frequencies. The amplitude and phase of these vibrations were analyzed using the AFM system’s integrated lock-in amplifiers. As illustrated in Figure 1, the probe is exposed to two excitation mechanisms: (i) The piezoelectric driver PD-I induces sinusoidal vibrations at a frequency significantly below the cantilever’s first eigenmode frequency, typically in the range of 0.5–2 kHz on our instrument. This action propels the probe to engage and withdraw with the sample surface periodically, forming a force curve. The peak force, identified within this curve, is utilized as a feedback parameter. By sustaining a constant peak force between the probe and the sample, the topography of the sample can be obtained. Quantitative mechanical properties of the measurement points can be calculated using the contact mechanics models [18,22]. (ii) Concurrently, the piezoelectric actuator PD-II excites the probe at its higher eigenmode, inducing a minor vibration with an amplitude generally beneath 1 nm. This high-eigenmode oscillation amplitude remains considerably smaller than the PFT amplitude and can be varied independently.

### Signal routing and processing

The composite deflection signal from the photodetector, comprising both the low-frequency PeakForce component and the high-frequency eigenmode oscillation, is electrically split into two independent channels after the pre-amplifier for dedicated, parallel processing of PFT feedback path and high-eigenmode detection path.

The channel of the PFT feedback path is processed by the system’s built-in PeakForce electronics. A low-pass filter (200 kHz cut-off) and synchronization algorithms suppress high-frequency noise and baseline drift, ensuring stable force feedback for accurate topography and quantitative mechanical mapping. These processing routines are factory-implemented and operate transparently to the user [23].

The unfiltered signal branch of the high-eigenmode detection path is routed directly to the internal lock-in amplifiers. The high bandwidth of the photodetector (~50 MHz) and the lock-in amplifiers (5 MHz) preserves the full fidelity of the high-frequency eigenmode signals. The lock-in amplifiers demodulate the amplitude and phase at the specific excitation frequency of the higher eigenmode, providing the signals used for compositional contrast.

This combination of dual-frequency excitation and parallel signal processing enables the simultaneous, effective acquisition of quantitative quasi-static force maps and qualitative eigenmode-derived material contrast.

### Cantilevers and calibration procedures

Two distinct types of AFM probes were used to validate the universality of the method, namely, a soft probe (ScanAsyst-Air) and a stiff probe (NSC15/Al BS). The key calibrated parameters for each probe are summarized in Table 1.

**Table 1:** Calibrated cantilever parameters and experimental excitation frequencies.

Probe type	*k* (N·m^−1^)	*f*_2_ (kHz)	*f*_3_ (kHz)

ScanAsyst-Air	0.48	494.7	1218.1
NSC15/Al BS	33.35	1930.7	N/A

### Calibration of optical lever sensitivity and spring constant

The inverse optical lever sensitivity (InvOLS) and the spring constant (*k*) of each cantilever were calibrated following a standard procedure, which is built into the Bruker Nanoscope software. Briefly, InvOLS (in nm·V^−1^) was determined by averaging the slope of the constant compliance region from at least five force–distance curves acquired on a rigid sapphire surface. The standard deviation of these measurements was consistently less than 5%. Subsequently, the spring constant was calibrated using the thermal tune method. The cantilever was retracted more than 5 μm from the sample surface to record its thermal vibration power spectral density. A fit to the fundamental resonance peak, based on the equipartition theorem, yielded the spring constant.

#### Characterization of higher eigenmodes

The resonant frequencies of the higher eigenmodes were characterized by performing a frequency sweep using piezoelectric actuator PD-II while the tip was retracted approximately 200 nm from the surface to avoid any influence of tip–sample interactions. The amplitude–frequency response was measured using the built-in lock-in amplifiers, and the operational excitation frequencies were determined by referencing the peaks of these response curves. The free oscillation amplitude for the higher eigenmode (e.g., 600 pm) was set based on the amplitude–frequency response curve. The voltage amplitude measured by the lock-in amplifier was converted to nanometers using the InvOLS coefficient obtained from the calibration procedure described above.

#### Sample preparation

The MMT nanosheets in the form of a purified powder were provided by the research group of Prof. Yinan Hao at Inner Mongolia Agricultural University. The powder was synthesized through an ultrasonic exfoliation process (240 W, 90 min) detailed in their patent [24]. For AFM characterization, an aqueous suspension was prepared from this pre-exfoliated powder at a concentration appropriate for AFM imaging, approximately 0.1 mg·mL^−1^. This suspension was treated with mild ultrasound (bath sonication, 15 min) to ensure thorough dispersion. Subsequently, a 20 μL aliquot was drop-cast onto a clean SiO_2_/Si substrate and allowed to dry under ambient conditions.

#### Imaging parameters

All critical imaging parameters for the data presented in the figures are summarized in Table 2. The free oscillation amplitudes for the higher eigenmodes were selected independently under our experimental conditions to optimize the phase contrast while minimizing interference with the concurrent PeakForce tapping quantitative mechanical measurements. The higher eigenmode phase images from the soft ScanAsyst-Air probe contained a regular, instrument-derived artifact. To remove this non-sample-related contribution, a 3×3 median filter was applied, and all subsequent quantitative analysis was performed on the processed data.

**Table 2:** Summary of critical AFM imaging parameters.

Parameter	Figure 3 (ScanAsyst-Air)	Figure 4 (NSC15/Al BS)

PeakForce tapping		
PeakForce setpoint	0.4 nN	14 nN
PeakForce amplitude	50 nm	50 nm
PeakForce frequency	2 kHz	2 kHz

high eigenmode excitation		
eigenmode used	second/third	second
free amplitude	~280 pm/~600 pm	~800 pm
excite frequency	494.7 kHz/1218.1 kHz	1930.7 kHz

tapping mode (for Figure 4)	N/A	–
drive frequency	–	*f*₀ = 303.2 kHz
setpoint amplitude	–	17 nm (*A*/*A*₀ = 0.7)

## Results and Discussion

### Vibration control strategy in PFT-based multifrequency AFM mode

**Figure 2:**
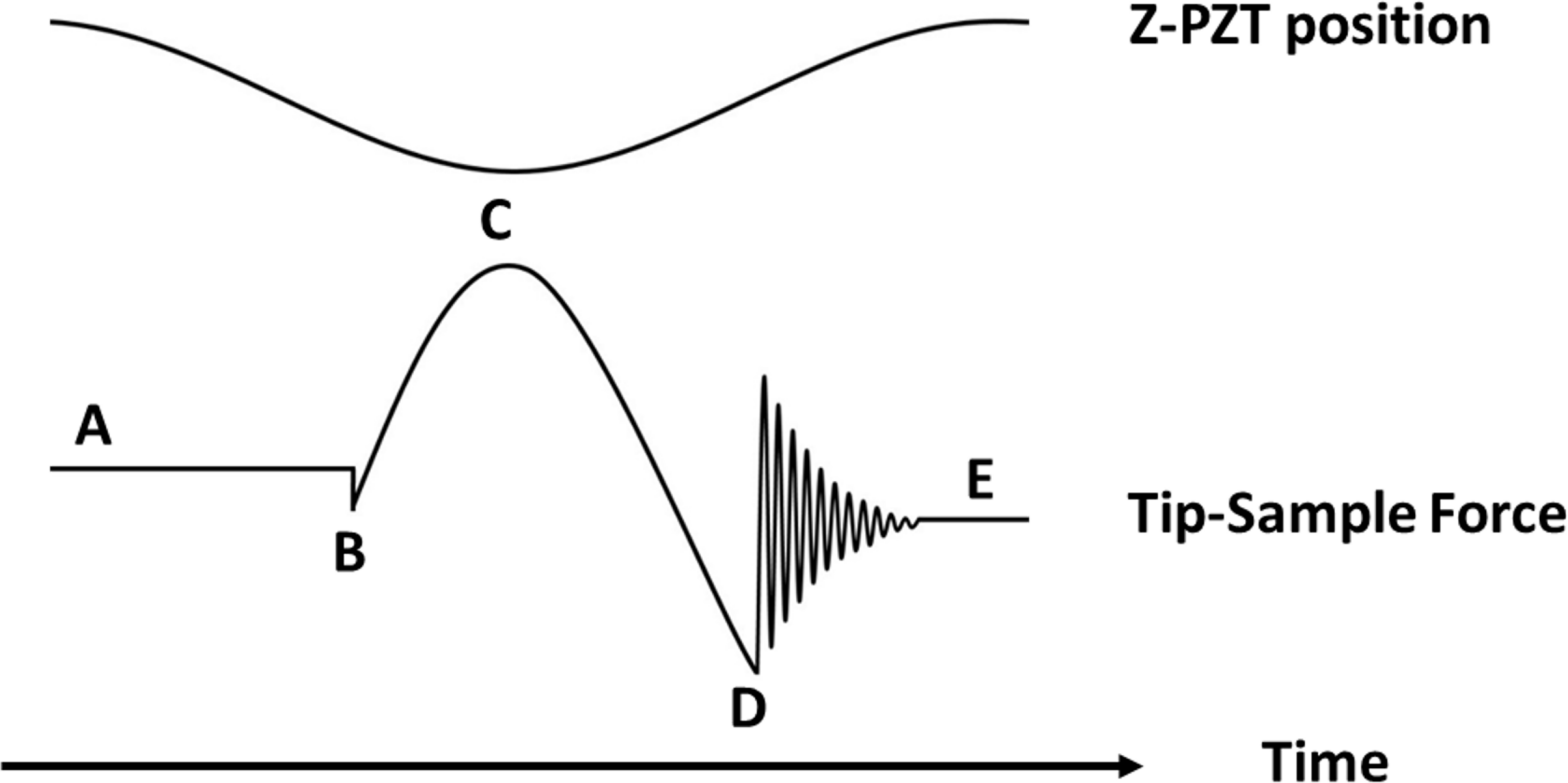
Driving and response signals in standard PFT mode. (Top) Vertical motion trajectory of the Z-piezo actuator (Z-PZT). (Bottom) Corresponding tip–sample force response, indicating key stages: (A) cycle start; (B) initial contact; (C) peak force; (D) detachment with adhesion peak; (Post-D) parasitic vibrations from the fundamental eigenmode; (E) cycle end.

In standard PFT mode, acquiring high-frequency force curves (typically 0.5–2 kHz on our instrument) induces cantilever oscillations at the fundamental eigenmode frequency. These oscillations persist as decaying vibrations after tip–sample detachment (Figure 2D). To avoid interference from this parasitic vibration, we employed higher eigenmodes (i.e., second and third modes) to excite the probe in our innovative multifrequency AFM mode.

The efficacy of this approach relies on two synergistic mechanisms. First, when the probe contacts the sample surface, tip–sample interaction forces induce a rapid resonance frequency shift and amplitude attenuation in the higher eigenmodes (Figure 2B–D). With the free vibration amplitude of the eigenmode being low, this inherent damping mechanism ensures that superimposed vibrations do not significantly perturb the peak force magnitude during tip–sample interaction. Second, the dedicated signal processing scheme detailed in Section “Experimental setup” effectively separates the composite cantilever deflection signal into two independent channels. The low-frequency path secures stable topographic feedback, while the high-frequency path preserves the eigenmode signals for material-sensitive detection.

This combination of physical damping and electronic signal separation enables simultaneous, effective acquisition of quasi-static force maps and eigenmode-derived material contrast.

### Dual-probe comparative analysis of MMT nanosheets

**Figure 3:**
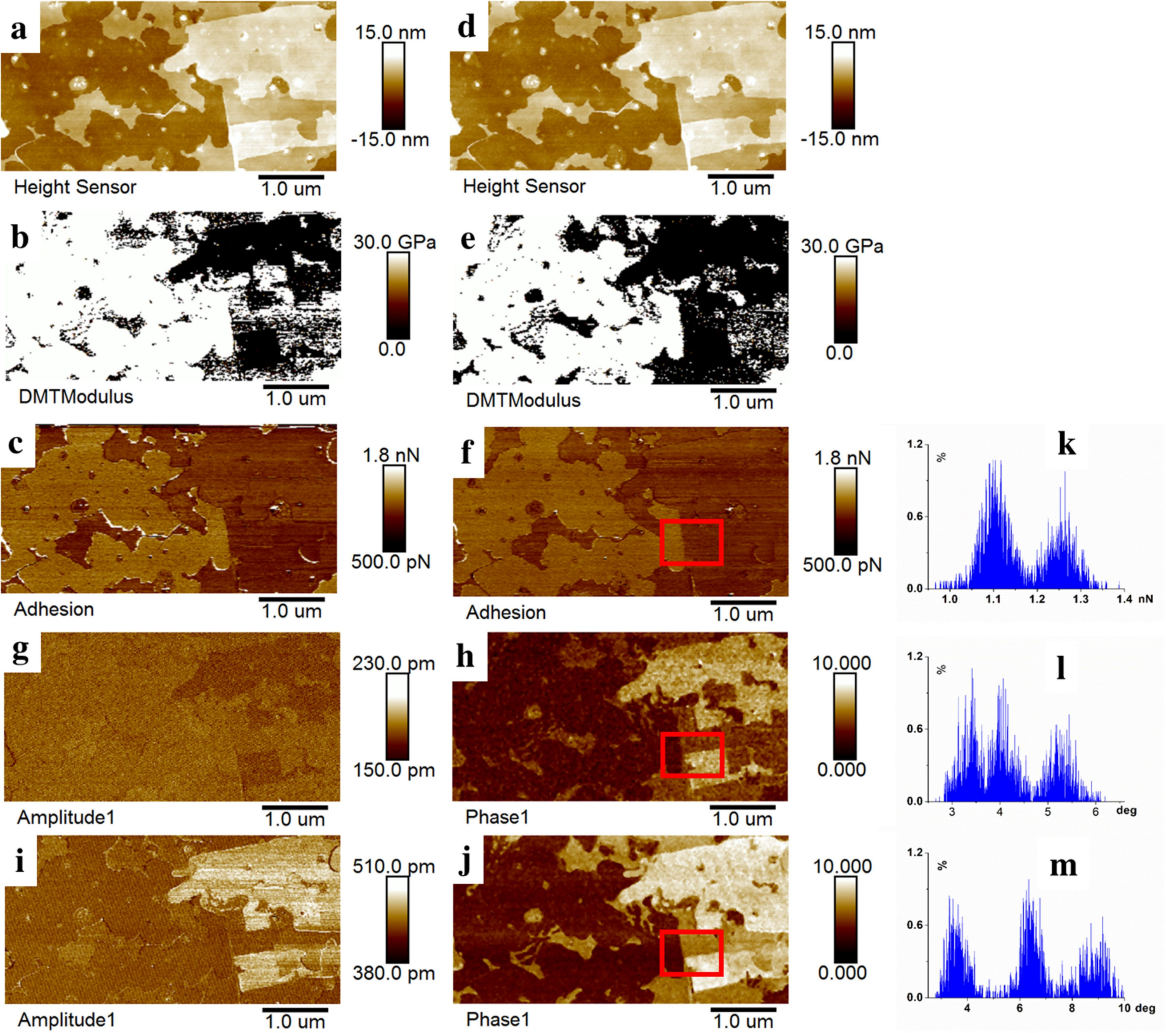
Surface property analysis of MMT nanosheets using the ScanAsyst-Air probe. (a–c) Topography, Young’s modulus, and adhesion force maps acquired through standard PFT mode. (d–h) Topography, Young’s modulus, adhesion force, second eigenmode amplitude and phase maps acquired through PFT+2nd method. (i, j) Third eigenmode amplitude and phase maps acquired through PFT+3rd method. (k–m) Histograms of data corresponding to the regions of interest in Figure 3e,f,h, and j.

The effectiveness of the method was evaluated using the soft (ScanAsyst-Air, 0.48 N·m^−1^) and stiff (NSC15/Al BS, 33.35 N·m^−1^) probes. Figure 3 presents results obtained with the ScanAsyst-Air probe under three operational modes, namely, standard PFT mode, PFT with superimposed second eigenmode vibration (PFT+2nd), and PFT with superimposed third eigenmode vibration (PFT+3rd). Due to the probe’s low stiffness, insufficient sample deformation led to significant variability in modulus measurements (Figure 3b), indicating the ScanAsyst-Air probe was unsuitable for reliable Young’s modulus quantification. Comparative analysis revealed a high degree of similarity in the topography, modulus, and adhesion contrast between the standard PFT (Figure 3a–c) and the PFT+2nd methods (Figure 3d–f). A consistent pattern was observed for the PFT+3rd mode across the measured properties; therefore, no additional images are displayed.

Amplitude and phase diagrams from the superimposed second and third eigenmode vibrations are shown in Figure 3g,h and Figure 3i,j, respectively. Both signals effectively differentiated MMT nanosheets from the substrate, with phase images exhibiting superior contrast. Histograms (Figure 3k–m), derived from selected regions in Figure 3c,h,j, further highlight the enhanced contrast in phase images compared to adhesion maps. Notably, the third eigenmode phase image provided the highest contrast, enabling clear distinction between thinner and thicker nanosheet regions.

**Figure 4:**
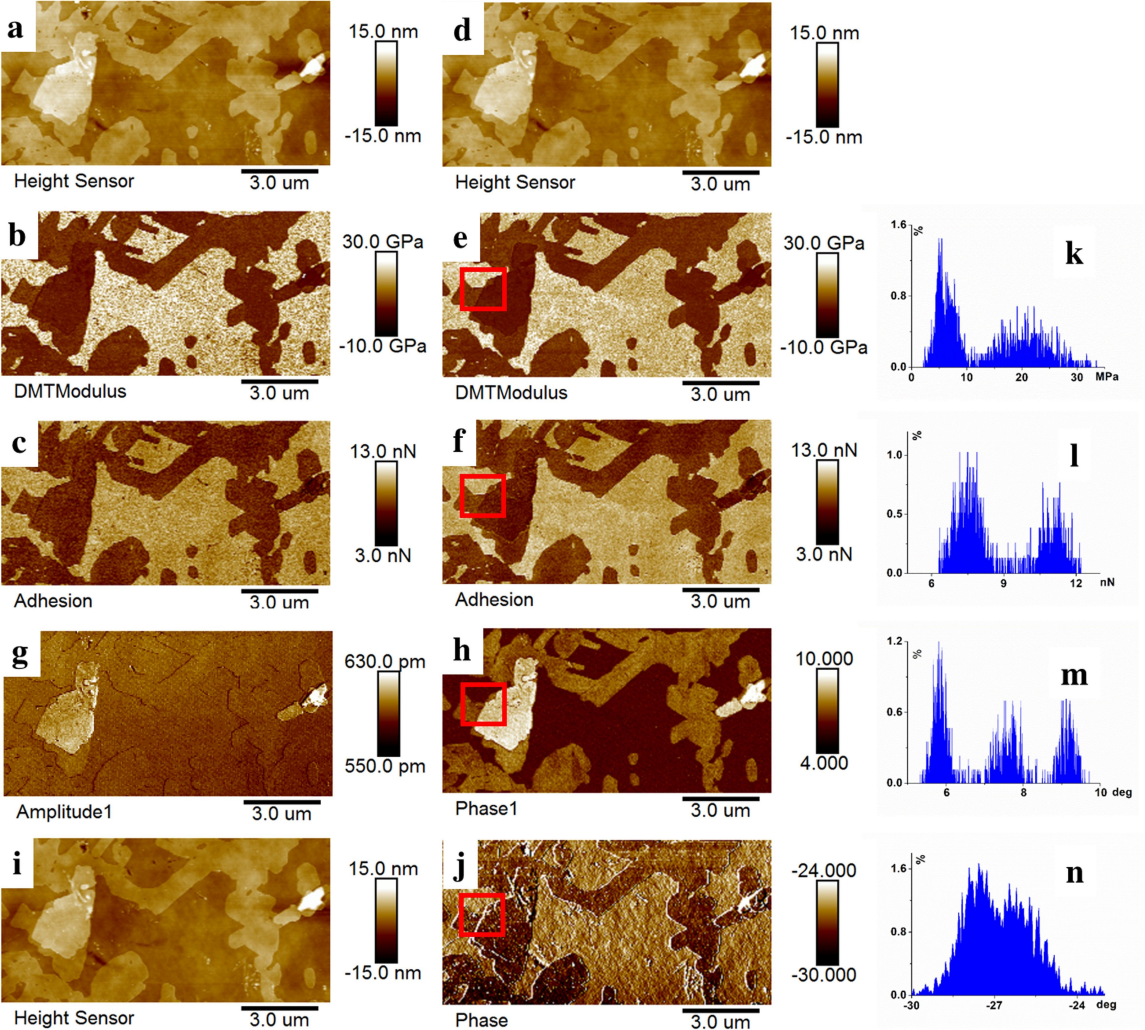
Surface property analysis of MMT nanosheets using the NSC15/Al BS probe. (a–c) Topography, Young’s modulus, and adhesion force maps acquired through standard PFT mode. (d–h) Topography, Young’s modulus, adhesion force, second eigenmode amplitude and phase maps acquired through PFT+2nd method. (i, j) Topography and phase maps acquired through standard tapping mode. (k–n) Histograms of data corresponding to the regions of interest in Figure 4e, f, h, and j.

For the NSC15/Al BS probe (Figure 4), the lock-in amplifier detected the first two eigenmodes. Measurements were performed in standard PFT mode (Figure 4a–c), PFT+2nd mode (Figure 4d–h), and conventional tapping mode (Figure 4i,j) at the same location. Similar to the soft probe, a high degree of similarity was observed in the topographic, modulus, and adhesion contrast between the standard PFT and PFT+2nd modes. Histograms in Figure 4k–n demonstrate that second eigenmode phase maps offered superior contrast compared to modulus, adhesion, and tapping mode images, enabling clear differentiation between the substrate, thinner and thicker regions of the nanosheets.

Comparative imaging with a soft probe (ScanAsyst-air) and a stiff probe (NSC15/Al BS) demonstrates that our method yields closely matched topography (~4 nm step height between the thinner and thicker nanosheet regions), confirming the robust height fidelity of the present approach. Quantitative modulus maps exhibit pronounced probe-dependence; the soft probe yields unreliable values, whereas the stiff probe yields narrow and reliable distributions, underscoring the critical influence of force-constant selection on modulus accuracy. To quantify imaging contrast without introducing additional variables, pixels were extracted from the same thinner and thicker nanosheet regions used for the reported histograms, and Ashman’s *D* values were computed for both Young’s modulus and phase signals using the method described in [25] (Table 3). *D* > 2 indicates clear separation of distributions. Modulus maps reveal probe-dependence; the soft probe fails to provide reliable separation, while the stiff probe successfully discriminates (*D* ≈ 2.7). However, phase imaging with both probes enables effective differentiation between these regions, demonstrating that the eigenmode contrast is less dependent on probe stiffness compared to quantitative mechanics maps.

**Table 3:** Ashman’s *D* for quantitative contrast between thinner and thicker nanosheet regions.

Probe Type	Young’s modulus	second-mode phase	third-mode phase

ScanAsyst-Air	N/A^a^	3.54	7.17
NSC15/Al BS	2.71	7.30	N/A^b^

^a^value is not available due to insufficient reliable modulus data for ScanAsyst-Air probe; ^b^no detectable signal for the third eigenmode for NSC15/Al BS probe.

To assess potential sample damage, we re-imaged the scan areas after the multifrequency measurements. No discernible sample damage or topographic alterations were observed within the specific regions of interest (ROIs) used for contrast quantification after multifrequency testing (see Supporting Information File 1, Figure S1), confirming that the nanomechanical data and phase contrast extracted from these ROIs are reliable and were not compromised by topographic alterations. In addition, independent reproducibility tests were performed on multiple regions, and the measurement results were highly consistent across these different areas (see Supporting Information File 1, Figure S2).

### Mechanistic insights and operational advantages

The material contrast generated by the superposed eigenmode phase originates from the cantilever’s high sensitivity to the gradient of tip–sample interactions and energy dissipation processes [6,26], complementing the absolute force measurements obtained from the quasi-static PeakForce feedback loop. This dual-mechanism approach enables the detection of a broad range of near-surface properties, including elastic modulus, viscoelasticity, and dissipation, greatly enhancing material differentiation.

The material differentiation capability observed in this experiment stems from the complementary synergy between quasi-static PFT and high-eigenmode excitation. PFT provides a stable mechanical baseline and quantitative mechanical measurements through force feedback control, while the high-eigenmode oscillation acts as a highly sensitive sensor for detecting the aforementioned interaction gradients. Employing low oscillation amplitudes under modal vibration conditions is a well-established strategy that has been proven to further enhance sensitivity [26–28]. The underlying mechanism is that low-amplitude operation confines the tip motion to a more localized region, thereby increasing its sensitivity to gradients in short-range interaction forces, such as dissipative and viscoelastic responses. The large-amplitude PeakForce motion provides a stable mechanical baseline that prevents the high-frequency micro-oscillation from undergoing the “pull-in” phenomenon, where the tip suddenly collapses into the sample surface due to excessive attractive forces, thus avoiding associated imaging instabilities. The critical role of a stable mechanical baseline for enabling high-frequency vibrational measurements is also recognized in concurrent AFM techniques [29]. This mechanism enables the high eigenmode to stably and sensitively probe the near-surface region and detect a broader spectrum of tip–sample interactions, which contributes to improved material property contrast in phase and amplitude channels, thereby providing a critical complement to the quantitative yet averaged information obtained from quasi-static force curves.

The superior phase contrast of the third eigenmode observed in this experiment can be primarily attributed to its enhanced capability for probing near-surface interaction forces, as supported by studies on multimodal AFM [26]. The higher dynamic stiffness of the third eigenmode, compared to the second, makes it less susceptible to damping by long-range surface forces (e.g., van der Waals forces). This allows it to sense the sharper gradients of short-range interactions and dissipative processes within the sample’s near-surface region more effectively. Consequently, the phase signal resolves the more pronounced differences between thinner and thicker nanosheet regions with exceptional clarity.

Operationally, compared to conventional quantitative mechanical mapping, this dual-mode detection framework, integrating quantitative mechanical mapping via quasi-static force curves with qualitative contrast derived from eigenmode vibrations, demonstrates a key practical advantage, that is, reduced dependence on probe stiffness. Our experimental validation reveals that, while soft probes exhibit modulus quantification variability due to limited contact deformation (Figure 3b), their superimposed higher-order eigenmode signals provide complementary contrast, enabling reliable differentiation of thinner/thicker nanosheet regions. Conversely, stiff probes provide both reliable quantitative mechanics and high-contrast phase imaging. This novel methodology thus synergizes mechanical precision with sensitive qualitative discrimination, advancing nanoscale material characterization.

## Conclusion

This study successfully integrates PFT mode with eigenmode vibrations, establishing a novel multifrequency AFM technique. This method synergizes quasi-static force control with dynamic vibrational signals, enabling simultaneous high-resolution topography and mechanical mapping, as well as enhanced material contrast through eigenmode-derived phase imaging. Experimental results demonstrate that superimposing low-amplitude higher-eigenmode vibrations (e.g., second and third modes) does not significantly perturb core mechanical parameters (e.g., modulus and adhesion) in PFT mode, while providing additional channels for material differentiation.

The key innovation lies in its compatibility and robustness. By decoupling quasi-static force feedback from dynamic vibrational signals, the method broadens the applicable range of probe stiffness, overcoming the stringent requirements of conventional PFT mode. Soft probes (e.g., ScanAsyst-Air), despite generating modulus variability due to insufficient contact deformation, retain efficacy in material discrimination via eigenmode phase contrast. In contrast, stiff probes (e.g., NSC15/Al BS) not only enable reliable mechanical quantification but also achieve high-contrast phase imaging. Additionally, synchronized signal processing algorithms and low-pass filtering effectively suppress parasitic vibrations and baseline drift, ensuring sub-nanometer topographic resolution.

This method offers a practical new tool for characterizing heterogeneous nanomaterials, particularly in scenarios where probe selection is limited or multiparameter measurements are required. Future research should prioritize a systematic parameter optimization study to establish guidelines for balancing contrast enhancement with measurement fidelity across a wide range of material systems. A particular focus should be on understanding the influence of eigenmode selection, excitation parameters, and their interplay with PeakForce settings on the reproducibility of material contrast, which is key to accelerating the practical adoption of this technique.

## Supporting Information

File 1 Post-measurement topography verification and reproducibility tests on multiple sample regions.

## Data Availability

Data generated and analyzed during this study is available from the corresponding author upon reasonable request.
