# Supplementary material for: Multifrequency AFM integrating PeakForce tapping and higher eigenmodes for heterogeneous surface characterization
Source: Beilstein J Nanotechnol. 2025 Nov 17;16:2077–85. doi: 10.3762/bjnano.16.142 (PMC12642942; doi:10.3762/bjnano.16.142)
Supplement: File 1 — Post-measurement topography verification and reproducibility tests on multiple sample regions. [file Beilstein_J_Nanotechnol-16-2077-s001.pdf]

BEILSTEIN JOURNAL OF NANOTECHNOLOGY

Supporting Information

for

Multifrequency AFM integrating PeakForce tapping and higher eigenmodes for heterogeneous surface characterization

Yanping Wei, Jiafeng Shen, Yirong Yao, Xuke Li, Ming Li and Peiling Ke

Beilstein J. Nanotechnol. **2025,** *16,* 2077–2085. doi:10.3762/bjnano.16.142

Post-measurement topography verification and reproducibility tests on multiple sample regions

License and Terms: This is a supporting information file under the terms of the Creative Commons Attribution License (https://creativecommons.org/licenses/by/4.0). Please note that the reuse, redistribution and reproduction in particular requires that the author(s) and source are credited and that individual graphics may be subject to special legal provisions.
The license is subject to the *Beilstein Journal of Nanotechnology* terms and conditions: (https://www.beilstein-journals.org/bjnano/terms)

Assessment of Topographic Integrity After Multi-Frequency AFM Measurement

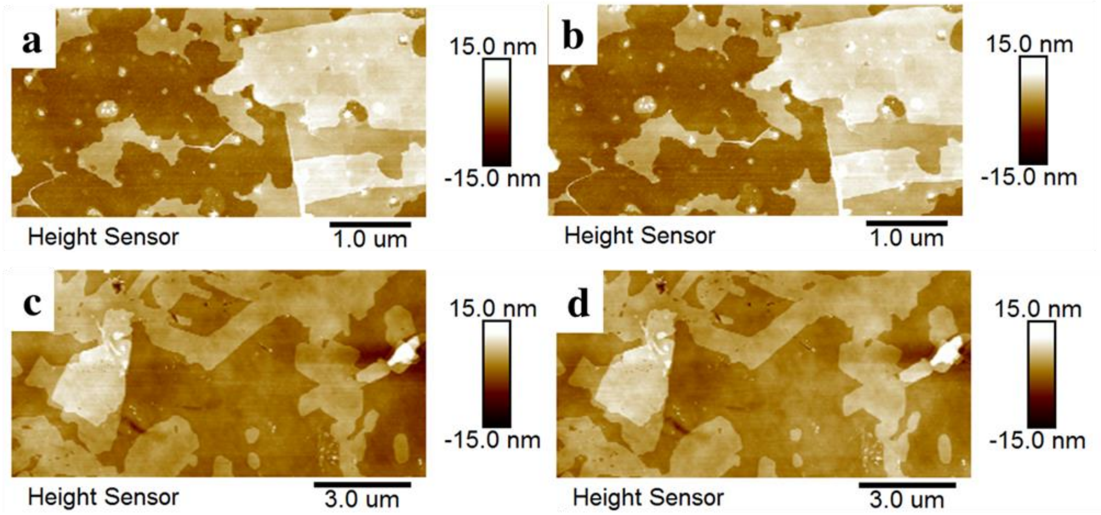

Figure S1: Topographic integrity of the scan areas used in the main text, before and after multi-frequency testing. No discernible sample damage or topographic alterations are observed within the specific regions of interest used for contrast quantification (outlined in Figure 3 and 4 of the main text), confirming that the nanomechanical and phase data extracted from these areas are reliable. (a, b) Topography of the scan area shown in Figure 3 of the main text: (a) initial scan and (b) final re-scan after multi-frequency measurement. (c, d) Topography of the scan area shown in Figure 4 of the main text: (c) initial scan and (d) final re-scan after multi-frequency measurement.

Reproducibility of Eigenmode Phase Contrast in PeakForce Tapping-Based Multifrequency Mode

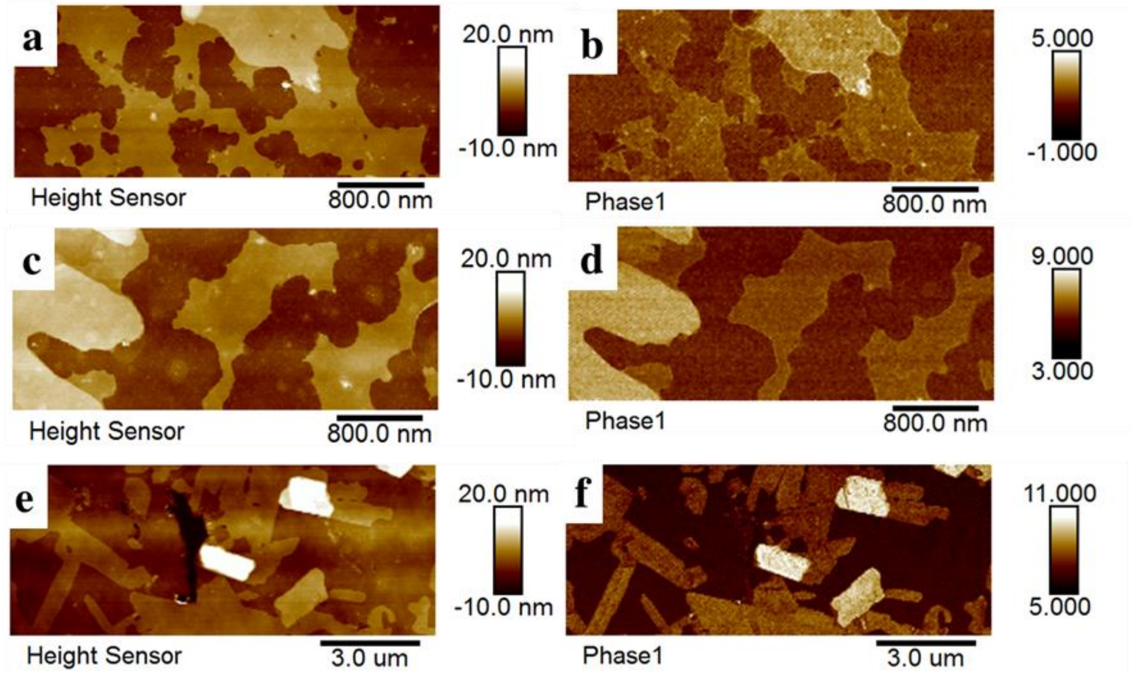

Figure S2: Reproducibility tests of eigenmode phase contrast acquired in the multi-frequency mode (PeakForce tapping with superimposed higher eigenmode excitation) on independently scanned sample regions. (a, b) Topography and corresponding 2nd-eigenmode phase image acquired with the soft Scanasyst-air probe on one region. (c, d) Topography and corresponding 3rd-eigenmode phase image acquired with the soft Scanasyst-air probe on a separate region. (e, f) Topography and corresponding 2nd-eigenmode phase image acquired with the stiff NSC15/Al BS probe on a third region. Ashman's D values for the contrast between thinner and thicker nanosheet regions, calculated from the phase images (b, d, f), are 3.12 (Scanasyst-air, 2nd-mode), 6.96 (Scanasyst-air, 3rd-mode), and 6.91 (NSC15/Al BS, 2nd-mode), respectively. These results are consistent with the trends reported in the main text (see Table 3 of the main text). The regions presented in these panels were selected by excluding areas where

the origin of the phase contrast was ambiguous, thereby focusing the validation of the method's core capability on unambiguous cases.
